# Climate

**Published:** 1858-07

**Authors:** 


### THE EPITOME OF SANITARY LITERATURE.

If the amount of literature supplied on any given subject or science may be received as an indication of national progress on such subject, it is obvious that sanitary science is rapidly advancing in these days. Our book list will show how large an addition to sanitary literature has this year been made.

Climate. Dr. Edwin Lee * has a small but neat work, entitled, *The Effect of Climate on Tuberculous Disease.* It contains the subject matter of an essay, for which the "Fiske Fund Prize" was awarded. Speaking of the treatment of consumption, this writer observes, while advocating the peremptory necessity of prescribing air, that an equable climate is not the most desirable, and gives notices of the following most frequented places of resort for consumptives:—Pau, Hyeres, Nice, Menton, Pisa, Rome, Naples, Palermo, Malta, Madeira, Malaga, Algiers, and Egypt. In these notices, he supplies important information relative to geographical position, climate, amusements, accommodation, etc. He is of opinion that the climate of Upper Egypt is more than any other calculated to render service to a large proportion of invalids from phthisis in the early stages.

* The Effect of Climate on Tuberculous Disease, with an Appendix. By Edwin Lee, M.D. London: Churchill. 1858.

